# The association between nation-level social and economic indices and suicide rates: A pilot study

**DOI:** 10.3389/fsoc.2023.1123284

**Published:** 2023-03-31

**Authors:** Ravi Philip Rajkumar

**Affiliations:** Department of Psychiatry, Jawaharlal Institute of Postgraduate Medical Education and Research, Puducherry, India

**Keywords:** suicide, sustainability, gender, social capital, economic inequality, gender inequality, subjective wellbeing

## Abstract

Ever since the pioneering work of Emile Durkheim, it has been known that regional or national suicide rates can be influenced by a variety of social and economic factors. Recent research has found a robust association between two country-level economic indices—gross national product and unemployment rate—and suicide rates, particularly in men. However, the association between other country-level social indices—such as measures of social integration, inequality, environmental preservation and political freedom—and suicide rates has not been studied at the cross-national level. In the current study, national suicide rates for men and women were examined in relation to seven indices measuring subjective wellbeing, sustainable development, type of political regime, economic and gender inequality, and social capital. It was found that the Happy Planet Index, a composite measure of subjective wellbeing and sustainable development, was negatively associated with suicide rates independent of gender, and even after adjusting for possible confounding factors. Economic inequality was associated with suicide in men, and social capital was associated with suicide in women. Moreover, the strength and direction of the associations observed between socioeconomic indices and suicide varied across income groups. These results highlight the need for a closer evaluation of the link between large-scale (“macro”) social factors and individual (“micro”) psychological factors, as well as the importance of integrating these factors into suicide prevention programmes at the national level.

## Introduction

Suicide is one of the leading causes of premature mortality worldwide. An analysis of global suicide data for the period 1990–2016, covering 195 countries, found that suicide was among the ten leading causes of years of life lost in Europe, the Americas and the Asia-Pacific region (Naghavi, 2019). Because of this, suicide prevention has been accorded one of the highest priorities in national and international public health plans, such as the World Health Organization's Comprehensive Mental Health Action Plan (World Health Organization, 2021a). The act of suicide is a complex behavior which results from the interaction between an innate diathesis and external stressors (van Heeringen, 2012). However, this interaction cannot be divorced from the social context in which it takes place (Mueller et al., 2021). In other words, suicide is best understood from a biopsychosocial perspective, with due weightage given to individual genetic variants, exposure to specific forms of stress, the biological and psychological processes mediating gene-environment interactions, and the individual victim's broader social, cultural and economic context (Staples and Widger, 2012; Duprey et al., 2021; Edwards et al., 2021).

Studies of the relationship between socioeconomic factors and suicide date back to the pioneering work of Emile Durkheim, who defined four subtypes of suicide on sociological grounds in his monograph “On Suicide” in 1897 (Durkheim, 2006). Of these subtypes, the “egoistic” suicide reflects a lack of integration of the individual into the community; the “anomic” type arises from periods of social or economic turmoil; the “fatalistic” type results from societal over-regulation; and the “altruistic” type results from the subordination of the individual's needs to those of a broader group, or of society as a whole. Thus, the former two types represent an alienation or disconnection from society, while the latter two result from excessive social integration and control.

Though Durkheim's proposals were subsequently found to have significant limitations (Kushner and Sterk, 2005), they were influential both in terms of framing suicide as a sociological problem, and in terms of examining the relationship of suicide rates to broad measures of social and economic development and stability (Condorelli, 2016). Subsequent research has provided support for certain aspects of Durkheim's original formulation. For example, the relationship of “anomic” suicides to periods of social deregulation or disintegration has been demonstrated in countries undergoing such changes, such as the former Soviet Union (Pridemore et al., 2007). Likewise, the possibility of a relationship between a lack of social integration and “egoistic” suicides was recently verified in an analysis of data from 188 countries and territories, which found a significant negative correlation between cultural collectivism and suicide even after adjusting for confounders such as national wealth and religiosity (Pelham et al., 2022). However, *contra* Durkheim, studies of specific populations and regions have found that in some cases, social integration and “hyperregulation” are positively associated with suicide. Such suicides do not conform to Durkheim's category of “altruistic” suicide, but show a rough correspondence with the “fatalistic” category which Durkheim considered rare (Kushner and Sterk, 2005; Zayas and Pilat, 2008; Aliverdinia and Pridemore, 2009). A careful reading of the available evidence suggests that the relationship between social integration, social control and suicide is non-linear (Condorelli, 2016), and is significantly influenced by factors such as a country or region's prevailing culture, the degree of integration of the study population into the general population as a whole (e.g., the cases of migrants or ethnic minorities), and economic variables (Lester, 2001; Borum, 2014; Eskin et al., 2020).

The relationship between economic factors and suicide, though only partly related to Durkheim's theories, has yielded certain important findings. For example, a study of European countries found that a higher risk of poverty, more rapid industrial growth, and lower spending on healthcare were associated with national suicide rates (Ferretti and Coluccia, 2009). A larger study of 35 countries confirmed the association between lower healthcare spending on suicide, and also identified a link between unemployment rates and suicide, particularly in men (Milner et al., 2012). In the largest and most recent study of this kind, an analysis of data from 175 countries replicated the association between unemployment and suicide, particularly in middle-aged men. This study also identified a negative association between increase in gross domestic product and suicide, though the magnitude of this effect was modest (Meda et al., 2022). A positive association between female labor force participation and male suicides was also reported in one of these studies, providing further support for a link between unemployment and suicide that is more significant in men (Milner et al., 2012).

Apart from the measures discussed above, several other social and economic factors have been identified as contributing to suicide in specific groups or populations, and merit further investigation at a cross-national level. These include the following:

- Gender inequality, which has been associated with an increase in suicidal ideation in both genders; on the other hand, increases in gender equality with a country were associated with reduced female suicide rates, possibly resulting in an increased male-to-female suicide ratio (Assarsson et al., 2018; Chang et al., 2019; Milner et al., 2020). Gender equality also appears to exert a modest protective effect on the suicides associated with economic disruptions (Reeves and Stuckler, 2016).
- Economic inequality, which has been associated with an increased suicide risk (Shah, 2012; Machado et al., 2015). There is some evidence that this effect may be more pronounced in certain demographic groups, such as men (Fernquist, 2003; Gunnell et al., 2003), young adults aged 18–35 (Gunnell et al., 2003; Miller et al., 2005) and elderly individuals who are economically dependent on others (Shah, 2012; Rodriguez Ruzafa et al., 2022).
- Social capital, which is a measure of social integration and cohesion hypothesized to protect against suicide (Patel, 2010). Empirical evidence suggests that measures of social capital are negatively associated with suicide rates in the general population, though the magnitude of this effect is modest (Mignone and O'Neil, 2005; Kunst et al., 2013; Okamoto et al., 2013; Smith and Kawachi, 2014).
- Environmental sustainability, particularly in so far as it pertains to parameters such as exposure to “green spaces” which may be protective against suicide. Access to such spaces may be endangered by processes of urbanization and industrialization (Helbich et al., 2018; Jiang et al., 2021; Mendoza et al., 2023).
- The nature of a nation's government, more specifically whether it tends toward a more authoritarian or a more democratic structure. Some researchers have found an association between a decrease in authoritarian government and reduced suicide rates (Varnik et al., 1994) and between increased democratization and reduced suicide mortality (Mackenbach et al., 2013). However, others have found that this effect is significant only after controlling for economic factors (Makinen, 2000), and more recently, an association in the opposite direction has been noted in an analysis of data from 43 countries, in which suicide rates were positively associated with democracy (Marotta et al., 2019).

Subjective wellbeing (SWB) is a measure of an individual's perceived level of happiness (the affective component) and satisfaction with life (the cognitive component). Subjective wellbeing may be influenced by a wide range of social and economic factors, including cultural values (Steel et al., 2018; Germani et al., 2021), economic inequality (Ngamaba, 2017; Ugur, 2021), gender inequality (Batz-Barbarich et al., 2018; Li et al., 2021), social capital (Helliwell and Putnam, 2004), access to green spaces (Huerta and Utomo, 2021; Xu et al., 2022) and the political structure and orientation of a nation's government (Jorm and Ryan, 2014). SWB has been associated with a reduced risk of suicidal ideation and behavior in a wide range of contexts (Furlanetto and Stefanello, 2011; Lew et al., 2019; Jo and Kim, 2020), including at the cross-national level (Wu et al., 2013; Hsu et al., 2019; Qian, 2021). Given the relationship between SWB and socioeconomic factors on the one hand, and suicide on the other, it is possible that this variable may mediate, at least in part, the relationship between broader, “macro”-level social and economic factors and suicide.

In recent years, several indicators that attempt to capture the above factors have been designed by groups of experts, based on large cross-national data sets. The current study is a preliminary attempt to examine the associations between these indicators of social and economic wellbeing, subjective wellbeing at a national level, and national suicide rates, while adjusting for those economic factors that were significantly associated with suicide in earlier research. As a secondary objective, the possibility of a mediating role of SWB was also analyzed.

## Materials and methods

The current study is a cross-sectional, cross-national ecological association study. The primary objective of this study was to examine the associations between specified indices of social and economic performance and national suicide rates. The secondary objectives of this study were: (a) to examine the relationship between national subjective wellbeing and national suicide rates, and (b) to examine the possibility that subjective wellbeing mediates the association between socioeconomic index measures and national suicide rates.

### Data sources

Data on national suicide rates was obtained from the most recent World Health Organization publication, which was published in 2021 and provided estimated national suicide rates for 183 countries and regions, current as of the year 2019 (World Health Organization, 2021b). To examine the gender-specific effects of socioeconomic factors on suicide, both the total suicide rate and the specific suicide rates for men and women were entered separately into all analyses, and the ratio of male to female suicides for each country was computed. To minimize the confounding effect of age on national suicide rates, the age-standardized suicide rate was used instead of the crude suicide rate.

The following social and economic indices were examined in the current study:

- *The World Happiness Report score*, a measure of national subjective wellbeing, computed based on cross-national survey data by independent experts. Subjective wellbeing has been associated with lower suicide rates, but this association is often confounded by economic factors. Data on this variable was obtained from the World Happiness Report, 2019 (Helliwell et al., 2019).
- *The Happy Planet Index*, a composite measure that combines subjective wellbeing (a measure of personal happiness) and ecological footprint (a measure of sustainable development). Both subjective wellbeing and environmental factors have been associated with regional variations in suicide rates. Data on this variable was obtained from the Happy Planet Index website for the year 2019 (Happy Planet Index, 2019).
- *The Democracy Index*, a composite measure of the election process, government functioning, participation by citizens in politics, and individual liberty, calculated by the Economist Intelligence Unit. Government policies and civil liberty can influence suicide rates both directly and *via* economic intermediaries. Data on this variable was obtained from the Economist Intelligence Unit's publication for the year 2019 (Economist Intelligence Unit, 2019).
- *The Gender Development Index*, a composite measure of gender disparities in life expectancy, education and income. Gender inequality has been associated with gender differences in suicidal ideation and behavior. Data on this variable was obtained from the United Nations Development Programme (2019).
- *The Gender Inequality Index*, a composite measure of reproductive health, women's empowerment, and participation of women in the workforce. Data on this variable was also obtained from the United Nations Human Development Report, 2019 (United Nations Development Programme, 2019).
- *The Gini Coefficient of income inequality*, a statistical measure of the dispersion in levels of income across a country. Economic inequality may be positively associated with suicide rates at a regional level. Data on this variable was obtained from the World Bank's database (The World Bank, 2022).
- *The Legatum Index of Social Capital*, a composite measure of the strength of interpersonal relationships, trust and participation in public activities, and social values, compiled by the Legatum Institute. Social capital has been found to correlate negatively with suicide by some, but not all, researchers. Data on this variable was obtained from the Legatum Institute's database (Legatum Institute, 2020).

Data on all these variables was obtained for the year 2018–2019, as this was the last calendar year for which reliable data was available for most countries, and to minimize the confounding effects of the subsequent COVID-19 pandemic (2020–2022) on national suicide rates (Farooq et al., 2021).

To correct for the possible confounding effects of gross domestic product (GDP) per capita and unemployment, particularly in men (Meda et al., 2022), information on both these variables was obtained for the year 2018–19 from the World Bank's database (The World Bank, 2022). In addition, an attempt was made to control for certain other confounding factors that have been associated with regional or national suicide rates. These include the Human Development Index (HDI) (Khazaei et al., 2017; Yasir Arafat et al., 2022), land area and population density (Vichi et al., 2020; Jakobsen and Lund, 2022; Rostami et al., 2022), urbanization (Ivey-Stephenson et al., 2017; Li and Katikireddi, 2019), and distance from the equator (An et al., 2023). Information on the HDI for the year 2019 was obtained from the United Nations Human Development Report (United Nations Development Programme, 2019); data on land area and population density was obtained from the World Bank's Open Data database (The World Bank, 2020); and data on latitude was obtained for the capital city of each included country from Google Earth (Google, 2023).

### Data analysis

All study variables were tested for normality prior to analysis. National suicide rates, gross domestic product per capita, and national unemployment rates did not conform to a Gaussian distribution (*p* < 0.01, Shapiro-Wilk test) and were converted to an approximately Gaussian distribution using a natural logarithmic transformation prior to analysis.

In the initial phase of the analysis, unadjusted bivariate correlations between national suicide rates (total, male, and female) and the seven selected social and economic indices were computed using Pearson's correlation coefficient (*r*). Subsequent to these, partial correlation coefficients (Pearson's partial *r*) were computed for the same variables, while controlling for the confounding factors enumerated above. All these analyses were two-tailed, and the significance level was set at *p* < 0.05. Unadjusted bivariate correlations were also subjected to Bonferroni's correction for multiple comparisons, to minimize the risk of false-positive associations. To examine if this association varied across income groups, these analyses were conducted both for the entire dataset of 183 countries, and for the four income sub-groups (upper, high middle, low middle and low) specified by the World Bank's income group classification (The World Bank, 2020).

In the next step, indices associated with each suicide rate (total, male and female) at *p* < 0.2 or less were entered into a multivariate linear regression model, to confirm and quantify the strength of the association between these variables and national suicide rates. Variance inflation factors (VIF) were computed for each linear regression analysis. If the VIF was >4 for any variable, indicating possible multicollinearity, the analysis was repeated after excluding the concerned variable.

Mediation analyses using Sobel's test were carried out where indicated, based on the results of the bivariate tests, to examine whether subjective wellbeing mediated the relationship between large-scale socioeconomic indicators and suicide rates.

Scatter plots for the seven indices of interest were also visually inspected to examine the possibility of a non-linear association between these indices and suicide rates. If such a pattern was suggested by the observed curve, non-linear associations (logarithmic, quadratic and cubic) were examined using the curve fitting function of the Statistical Package for Social Sciences, version 26.0 (SPSS v26.0).

## Results

A total of 183 countries were included in the analysis. The median age-standardized suicide rate across all countries was 8.3 per 100,000 population, with an inter-quartile range (IQR) of 7.15. The median male suicide rate was 13.3 per 100,000 population (IQR 12.25), while the median female suicide rate was 3.8 per 100,000 population (IQR 3.9). A summary of descriptive statistics for all countries included in this study is provided in Table 1.

**Table 1:** Descriptive statistics for all indices included in the current study.

**Variable**	**Number of countries for which data was available**	**Normally distributed?**	**Mean (SD)/ median (IQR)**	**Maximum value**	**Minimum value**
Suicide rate per 100,000 population	183	No	8.30 (7.15)	87.5 (Lesotho)	0.3 (Antigua and Barbuda)
Male suicide rate	183	No	13.30 (12.25)	146.9 (Lesotho)	0.0 (Antigua and Barbuda)
Female suicide rate	183	No	3.80 (3.90)	34.6 (Lesotho)	0.2 (Barbados)
Male-to-female suicide ratio	183	No	3.35 (1.93)	0.0 (Antigua and Barbuda)	13.42 (Solomon Islands)
World happiness report score	151	Yes	5.39 (1.13)	7.77 (Finland)	2.85 (South Sudan)
Happy planet index	149	Yes	44.59 (8.27)	62.10 (Costa Rica)	24.30 (Qatar)
Democracy index	164	Yes	5.43 (2.25)	9.87 (Norway)	1.08 (North Korea)
Gender development index	165	Yes	0.94 (0.08)	1.04 (Latvia)	0.49 (Yemen)
Gender inequality index	162	Yes	0.34 (0.19)	0.80 (Yemen)	0.03 (Switzerland)
Gini coefficient of income inequality	163	Yes	37.68 (7.94)	63.00 (South Africa)	23.20 (Slovakia)
Legatum index of social capital	165	Yes	52.70 (9.07)	77.24 (Denmark)	22.32 (Afghanistan)
Gross domestic product per capita	182	No	5,200.25 (14,864.80)	135,682.80 (Luxembourg)	236.80 (Burundi)
Unemployment rate	178	No	6.40 (6.53)	33.60 (South Africa)	0.30 (Qatar)
Human development index	181	No	0.726 (0.236)	0.954 (Norway)	0.377 (Niger)
Land surface area	183	No	143,350.00 (539,640.00)	1,638,000.00 (Russian Federation)	300.00 (Maldives)
Total population	183	No	9,771.14 (28,805.31)	1,408,000,000 (China)	92,120 (Antigua and Barbuda)
Population density	183	No	83.01 (133.26)	8,322.69 (Singapore)	2.18 (Mongolia)
Urbanization	183	No	59.00 (36.50)	100.00 (Kuwait, Singapore)	13.00 (Papua New Guinea)
Latitude	183	No	N/A	N/A	N/A

IQR, inter-quartile range; N/A, not applicable; SD, standard deviation. Values are given as mean (SD) for normally distributed variables and median (IQR) for variables not following a normal distribution.

### Correlations between socioeconomic indices and national suicide rates

Bivariate correlations between social and economic indices and national suicide rates, both total and gender-specific, are presented in Table 2. An examination of the unadjusted correlations reveals that the Happy Planet Index is significantly and negatively correlated with national suicide rates irrespective of gender, and that this correlation remains significant after correction for multiple comparisons. However, the magnitude of this correlation was modest (|*r|* = 0.33–0.37). In addition, a weak positive correlation was observed between the Gini coefficient and the suicide rate in males, but this was not significant after correction for multiple comparisons.

**Table 2:** Adjusted and unadjusted bivariate correlations between social and economic indices and suicide rates.

**Variable**	**Total suicide rate**		**Male suicide rate**		**Female suicide rate**	
	**Unadjusted**	**Adjusted**	**Unadjusted**	**Adjusted**	**Unadjusted**	**Adjusted**
World happiness report score	−0.09 (0.277)	−0.02 (0.791)	−0.09 (0.267)	−0.02 (0.821)	−0.14 (0.091)	−0.01 (0.954)
Happy planet index	–0.37 (<0.001)^**^	–*0.30 (<0.001)*	–0.33 (<0.001)^**^	−0.26 (0.002)^**^	–0.37 (<0.001)^**^	−0.26 (0.002)^**^
Democracy index	0.08 (0.292)	0.15 (0.055)	0.11 (0.178)	0.18 (0.028)^*^	0.02 (0.802)	0.16 (0.051)
Gender development index	0.03 (0.705)	0.16 (0.043)	0.09 (0.254)	0.23 (0.004)^**^	−0.15 (0.059)	−0.01 (0.954)
Gender inequality index	0.02 (0.793)	−0.23(0.004)	0.02 (0.802)	−23 (0.004)^**^	0.05 (0.488)	−0.28 (<0.001)^**^
Gini coefficient of income inequality	0.14 (0.070)	0.13(0.104)	0.17 (0.029)^*^	0.17 (0.041)^*^	0.08 (0.324)	0.03 (0.707)
Legatum index of social capital	0.07 (0.393)	0.12 (0.138)	0.05 (0.495)	0.10 (0.218)	0.05 (0.548)	0.18 (0.023)^*^

All values are given as Pearson's *r* (significance level). “Adjusted” refers to partial correlations adjusted for gross domestic product (per capita), unemployment rate, Human Development Index, population density, urbanization and distance from the equator.

^*^Significant at *p* < 0.05; ^**^Significant at *p* < 0.01. Values in *italics* indicate statistically significant results after the use of Bonferroni's correction for a 10 × 10 correlation matrix.

When partial correlation coefficients were computed after adjusting for the GDP per capita, unemployment rate, Human Development Index, population density, urbanization and distance from the equator, it was observed that the associations noted for the Happy Planet Index were slightly attenuated, but remained significant at a similar level for all three suicide rates (|*r*| = 0.26–0.30). The total suicide rate also showed a weak positive correlation with the Gender Development Index (*r* = 0.16, *p* = 0.043). Adjusted values for the male suicide rate showed positive correlations with the Democracy Index (*r* = 0.18, *p* = 0.028), Gender Development Index (*r* = 0.23, *p* = 0.004) and Gini coefficient (*r* = 0.17, *p* = 0.041) and a negative correlation with the Gender Inequality Index (*r* = −0.23, *p* = 0.004). Adjusted values for the female suicide rate showed a positive correlation with the Legatum Index of Social Capital (*r* =0.18, *p* = 0.023) and, surprisingly, a negative correlation with the Gender Inequality Index (*r* = −0.28, *p* < 0.001). However, the strength of all these adjusted associations was weak (|*r|* < 0.3 for all associations) and they did not survive correction for multiple comparisons.

In unadjusted analyses, male-to-female suicide ratio was positively correlated with the Gender Development Index (*r* = 0.43, *p* < 0.001) and the Gini coefficient (*r* = 0.16, *p* = 0.04). Similar findings were obtained when these correlations were adjusted for GDP, unemployment, Human Development Index, population density, urbanization and distance from the equator (Gender Development Index: *r* = 0.43, *p* < 0.001; Gini coefficient: *r* = 0.20, *p* = 0.012). No other variables were significantly associated with the male-to-female suicide ratio.

Correlations between the confounding factors used in the partial correlation analyses and suicide rates are presented in Supplementary Table 1. In these analyses, it can be observed that both the Human Development Index and the population density are negatively correlated with suicide rates regardless of gender, while total land area is positively correlated with suicide rates. In addition, the GDP per capita is negatively correlated with the suicide rate in women. Supplementary Table 2 illustrates the fact that even though the socioeconomic indices used in this study show significant correlations with each other, none of these are suggestive of multicollinearity between indices. Correlations between confounding factors and the socioeconomic indices considered in this study are presented in Supplementary Table 3; the most significant finding in these analyses is the possibility of multicollinearity (|*r|* ≥ 0.8) between SWB and GII on the one hand, and per capita GDP and HDI on the other.

Supplementary Table 4 presents bivariate correlations between suicide rates and socioeconomic indices based on income groups. These analyses were carried out for 182 countries as the World Bank reported a lack of reliable data from one country (Venezuela) for the year 2019. It can be seen that the indices associated with national suicide rates differ significantly in certain key aspects between income groups. In low-income countries, social capital was positively associated with suicide for both genders, and income inequality was positively associated with suicide, particularly in men. In low middle-income countries, both SWB and the Happy Planet Index were negatively associated with suicide irrespective of gender. In high middle-income countries, a similar negative association with the Happy Planet Index was noted; in addition, both the Gender Development Index and social capital were positively correlated with suicide, both overall and in men. In high-income countries, there was a paradoxical positive correlation between SWB and the suicide rate in women. In this group, the Gender Inequality Index was negatively correlated with suicide rates, while the Democracy Index was positively correlated with suicide; neither of these associations was gender-specific.

### Linear regression analyses of variables associated with national suicide rates

The results of linear regression analyses for each national suicide rate (total, male, and female) are presented in Table 3. Variables were selected for inclusion in these analyses if they were significantly correlated with the suicide rate under consideration at *p* < 0.05 in the bivariate analyses.

**Table 3:** Multivariate linear regression analyses of socioeconomic indices associated with national suicide rates.

**Dependent variable**	**Variables entered into the model**	**Degrees of freedom**	**Model fitness statistic**	**Regression coefficients (β)**	**Significance level (*p*)**	**Variance inflation factors**	**Percentage of variance explained (adjusted R^2^)**
Total suicide rate	GDI	146	*F* = 7.76, *p* < 0.001	0.22	0.026^*^	1.73	18.80%
	HDI			−0.18	0.077	1.79	
	HPI			−0.37	<0.001^**^	1.19	
	Land			−0.04	0.674	1.8	
	PopDens			−0.15	0.136	1.77	
Male suicide rate	GDI	137	*F* = 8.89, *p* <0.001	0.27	0.009^**^	1.96	25.70%
	Gini			0.06	0.472	1.37	
	HDI			−0.15	0.184	2.41	
	HPI			−0.42	<0.001^**^	1.27	
	Land			−0.07	0.431	1.59	
	PopDens			−0.15	0.124	1.62	
Female suicide rate	HPI	146	*F* = 8.64, *p* <0.001	−0.36	<0.001^**^	1.19	20.70%
	HDI			−0.26	0.005^**^	1.57	
	Land			0.04	0.679	1.75	
	PopDens			−0.06	0.517	1.75	
	SocCap			0.27	0.003^**^	1.44	
Male-to-female suicide ratio	GDI	150	*F* = 15.29, *p* <0.001	0.4	<0.001^**^	1.05	22.20%
	Gini			0.2	0.008^**^	1	
	Pop			−0.13	0.076	1.05	

GDI, gender development index; Gini, gini coefficient of income inequality; HDI, human development index; HPI, happy planet index; Land, total land area; Pop, total population; PopDens, population density; SocCap, Legatum Index of Social Capital.

* Significant at *p* < 0.05;

** Significant at *p* < 0.01.

In the first model, the total suicide rate was the dependent variable. Six variables (HPI, GDI, GII, HDI, land area, and population density) were initially entered into the model. Due to multicollinearity between the GDI and GII, with a variance inflation factor (VIF) > 5, the latter variable was excluded. The final model, which included five variables, was statistically significant and explained around 19% of the variance in national suicide rates. Among individual variables, the HPI was negatively associated with suicide, while the GDI was positively associated with suicide.

In the second model, the suicide rate in men was the dependent variable. Seven variables (HPI, GDI, GII, Gini, HDI, land area, and population density) were initially entered, but the GII was excluded due to multicollinearity (VIF > 6). The final model, which included six variables, was statistically significant and explained around 26% of the variance in male suicide rates. Results for individual variables were similar to those observed for the total suicide rate.

In the third model, the suicide rate in women was the dependent variable. Seven variables (HPI, GII, Gini, social capital, HDI, land area and population density) were initially entered, but the GII was excluded due to multicollinearity (VIF > 5). The final model, which included six variables, was statistically significant and explained around 21% of the variance in female suicide rates. Results for individual variables revealed that the HPI and HDI were negatively associated with the female suicide rate, while the Legatum Index of Social Capital was positively associated with suicide in women.

In the fourth model, the male-to-female suicide ratio was the dependent variable. Three variables (GDI, Gini, and total population) were entered into the model. There was no evidence of multicollinearity between these variables. The final model was significant and explained around 22% of the variance in the male-to-female suicide ratio. Among individual variables, both the GDI and the Gini coefficient were positively associated with the male-to-female suicide ratio.

Separate linear regression analyses for each income group were not attempted due to the relatively low number of cases in each sub-group relative to the number of variables being studied.

### Mediation analyses

As can be seen from Table 2, the measure of SWB used in this study (the World Happiness Report score) was not significantly associated with the total suicide rate, male suicide rate, or male-to-female suicide ratio in bivariate analyses. Hence, mediation analyses were not considered for these dependent variables. In view of a trend-level association (*r* = −0.14, *p* = 0.091) between SWB and the suicide rate in women, mediation analyses were undertaken to examine if SWB mediated the association between the female suicide rate and the three socioeconomic indicators (Happy Planet Index, Gender Inequality Index and Legatum Index of Social Capital) that were themselves correlated with this outcome. The results of these analyses are presented in Table 4 and suggest that SWB does not mediate the association between broader social and economic indices and suicide rates in women.

**Table 4:** Mediation analyses between social and economic indices, subjective wellbeing, and the suicide rate in women.

**Variable**	**Regression coefficient (variable to female suicide rate)**	**Regression coefficient (variable to SWB)**	**Regression coefficient (SWB to female suicide rate)**	**Sobel test statistic**	**Significance level**
Gender inequality index	0.01 (0.02)	−0.14 (0.01)	0.01 (0.05)	−0.20	0.841
Happy planet index	−4.77 (0.98)	0.06 (0.01)	0.01 (0.05)	0.12	0.905
Legatum index of social capital	0.63 (1.05)	5.63 (0.50)	0.01 (0.05)	0.19	0.850

All regression coefficients are given as unstandardized regression coefficient (standard error).

SWB, subjective well-being.

### Possibility of a non-linear association between suicide rates and social and economic indices

Scatter plots for the association between suicide rates and the seven social and economic indices examined in this study are presented in the Supplementary material. As none of these was suggestive of a possible non-linear association, curve fitting was not attempted.

## Discussion

The suicide rate is an indicator of the psychological and social wellbeing of a population or community, and a reduction in this variable is a meaningful goal of social and economic development (World Health Organization, 2021a; da Costa et al., 2022). Though the factors involved in an individual suicide are complex and not easily amenable to linear analysis, variations in suicide at a regional or national level are likely to reflect differences in social and economic conditions (Mueller et al., 2021). Some of these factors, such as cultural values, economic prosperity and unemployment, have been identified by earlier researchers. The aim of this study was to extend this sociological perspective on suicide by examining the relationship between national suicide rates and selected indices of social and economic wellbeing. In broad terms, this study can be considered a preliminary attempt at identifying potential “macro”-level social and economic indicators that could be considered “social risk factors” for suicide.

### Sustainability and suicide

In the current study, the Happy Planet Index, which combines measures of individual wellbeing (subjective wellbeing and life expectancy) and sustainable development (ecological footprint) was negatively associated with national suicide rates for both genders, even after adjusting for confounders and correcting for multiple comparisons. This result remained significant in the partial correlation and linear regression analyses, suggesting that it is unlikely to be due to chance. Recent research has highlighted the associations between environmental factors and suicide risk: for example, air pollution is associated with suicide (Casas et al., 2017; Heo et al., 2021), while exposure to green spaces may exert a protective effect (Helbich et al., 2018; Jiang et al., 2021; Asri et al., 2022; Mendoza et al., 2023). There is also some evidence that rising temperatures, which may reflect anthropogenic climate change, are associated with a short-term increase in suicide rates (Heo et al., 2021; Casas et al., 2022). Though the exact mechanisms underlying these associations are uncertain, they raise the possibility of a meaningful link between environmental sustainability and suicide at the group level (Shen et al., 2022). Though the Happy Planet Index represents a promising attempt to develop a composite index of wellbeing and sustainability, it is only a partial measure of these complex factors as it includes only one measure of sustainability—the *per capita* ecological footprint. The United Nations Human Development Reports list a large number of additional measures of sustainability, such as fossil fuel consumption, fertilizer use, land degradation, and reductions in biodiversity (United Nations Development Programme, 2019). It is possible that the construction of a composite index of sustainable development, along the lines of the Human Development and Gender Development indices, may permit a better analysis of the relationship between environmentally sustainable development and suicide rates. For the moment, all that can be stated is that the Happy Planet Index, which reflects the balance between SWB and sustainable development, appears to be meaningfully and negatively associated with suicide rates, and this finding is consistent with the available evidence from published literature on links between environmental factors and suicide.

### Economic inequality and suicide

Economic inequality, as measured by the Gini coefficient, was positively associated with suicide rates in partial correlation analyses, particularly in men and in low-income countries. Though there is relatively little research on this association, the available evidence suggests that economic inequality is linked to suicide, and that certain groups, particularly young men and the elderly, may be more sensitive to such an effect (Gunnell et al., 2003; Shah, 2012). This effect may be less pronounced in high-income countries than in low- and middle-income countries (Leigh and Jencks, 2007; Campo-Arias and Herazo, 2015); this finding was also observed in the current study, in which the association between the Gini coefficient and suicide was strongest in low-income countries. There is some evidence to suggest that this association may be partially mediated by depression (Hong et al., 2011), though this could not be verified in the current study. Income inequality has also been associated with alcohol abuse, which is an additional risk factor for suicide (Auger et al., 2009). Research in individual subjects has found that men are more likely than women to develop depression and attempt suicide in the context of economic stressors (Quan and Arboleda-Florez, 1999; Alston, 2012), which may account for the gender difference observed in this study. The current results also suggest that efforts to alleviate economic inequality may be most effective at preventing suicide when they are implemented in low-income settings.

### Other social and economic indices and suicide

Though no other significant associations were identified in the unadjusted and regression analyses, the results of partial correlation analyses suggest a weak association between the Democracy Index and suicide in men, and between social capital and suicide in women. A prior study of forty-three countries found that suicide appeared to be more significant in democratic countries than in those with an authoritarian regime, particularly in emerging democracies. The reasons for this association are unknown, but may reflect “relaxations” in law enforcement and public order during the transition from an autocratic to a democratic regime, or the persistence of class divisions and economic inequalities in a nominally democratic country (Marotta et al., 2019). In sociological terms, some of these suicides can be understood as “anomic”, resulting from the disintegration of older traditions and institutions that have not yet been fully replaced by the structures associated with a democratic form of government (Lester, 1998a; Makinen, 2000; Kovacs, 2008). The association between social capital and suicide has been the subject of some controversy, with researchers arguing for both positive and negative associations (Carpiano and Kelly, 2005; Kushner and Sterk, 2005). From a sociological perspective, both these associations are explicable once it is understood that both excessive and deficient social integration and control can lead to suicide, and that the former may be particularly applicable to women (Iga et al., 1975; Johnson, 1979; Zhang, 2011). Such suicides may be particularly likely to occur in societies or cultures characterized by lower autonomy for women and a high emphasis on the honor/shame dichotomy (Aliverdinia and Pridemore, 2009; van Bergen et al., 2009). However, given certain limitations of the current study, both these gender-specific associations require replication.

In this study, the male-to-female suicide ratio was positively correlated with the Gender Development Index, though this index was not significantly associated with either the male or the female suicide rate. A possible explanation for this finding is that greater gender equality may slightly reduce the suicide rate in women, but not in men, skewing the ratio in favor of the latter (Milner et al., 2020; Cai et al., 2021); this explanation should be considered tentative and subject to correction based on further evidence. The male-to-female suicide ratio was also positively associated with economic inequality, which is a further pointer toward the increased vulnerability of men to this factor, as discussed in the previous section.

### Variations in the correlates of suicide rates across income groups

When the correlations between socioeconomic indices and suicide rates were analyzed for each of the World Bank's income groups, certain intriguing differences were noted between them. Thus, the Happy Planet Index was negatively associated with suicide in middle-income countries, but not in high- or low-income countries; the Gini index of economic inequality was positively associated with suicide in low-income countries, but not in middle- or high-income countries; the Gender Development Index was positively associated with suicide rates only in high middle-income countries; and the Democracy Index was positively associated with suicide only in high-income countries. These differential associations underline the importance of considering a country's level of economic prosperity, as well as its cultural background, when performing cross-national analyses. In low-income countries, where an inadequate income may affect access to even the basic amenities of life, such as food, shelter and healthcare, it is understandable that income inequality would correlate with psychological distress, hopelessness and suicide (Shayo and Lawala, 2019; Belete et al., 2021). In low middle-income countries, which are often subject to rapid urbanization, industrialization and economic development, it is likewise easy to see how subjective happiness, particularly when measured in a manner that yields multicollinearity with economic prosperity, could be negatively associated with suicide (McGuire et al., 2022). The association between a measure of gender development and suicide rates in high middle-income countries becomes comprehensible when one considers that most of the countries in this group are Asian, African or South American countries with high levels of cultural masculinity and traditionally well-defined gender roles. In such settings, moves toward greater gender equality and empowerment could lead to conflicts at the family and community levels, and perhaps to a sense of anomie in men, possibly leading to an increased risk of depression and suicide (Arrindell et al., 2003; Feigelman et al., 2021). Finally, the positive association between the Democracy Index and suicide in high-income countries could reflect the fact that many of the more “recent” members of this group of countries have undergone—or are undergoing—a transition from more authoritarian to more democratic forms of government, which has been associated with a temporary increase in suicide risk by earlier researchers (Marotta et al., 2019). While the aforementioned explanations are plausible and partly supported by the available evidence, they require careful replication, particularly in low- and middle-income countries (Bantjes et al., 2016).

### The mediating role of subjective wellbeing

The results of earlier research on the association between subjective wellbeing and suicide have been inconsistent. Though the majority of studies have found a negative correlation between these variables, some researchers have reported a positive correlation between the two, and others found no significant association between them (Lester, 1998b; Qian, 2021). A possible explanation for these variable results is that subjective wellbeing is only one aspect of positive mental health, and may not have any consistent effect on suicide: other dimensions of mental health, such as a sensecoherence or connectedness in life (Drum et al., 2017), may be more protective. Another explanation is that subjective wellbeing may partly mediate the association between broader socioeconomic factors and suicide. However, this hypothesis was not supported by the results of the current study, which failed to find evidence for a mediation effect. It is likely that other psychological factors, such as resilience (Cha and Lee, 2018) or a sense of meaning in life (Aviad-Wilchek and Ne'eman-Haviv, 2018), may mediate the association between social and economic variables and the risk of suicide. However, the current study was not designed to address these possibilities. It is also possible that this lack of a positive association arises from the manner in which SWB is operationalized. In the current study, the measure of SWB obtained from the World Happiness Report showed significant multicollinearity with both per capita GDP and the Human Development Index, suggesting that it may capture the material or economic aspects of wellbeing rather than the affective or relational aspects of this construct.

### Strengths and limitations

The strengths of this study are its reliance on large datasets, its coverage of low- and middle-income, its evaluation of factors that have not been studied extensively in relation to suicide, and its attempts to correct for possible confounders. However, certain important limitations of this work should be borne in mind. First, all analyses are based on country-level data, and cannot be extrapolated directly to individuals (the ecological fallacy); rather, they can only provide leads for further, more precise research in individuals. Second, there is a significant degree of uncertainty and under-reporting in national suicide rates, particularly in lower-income countries (Claassen et al., 2010), and this could affect the accuracy of the study results, leading to potential false-negative findings for certain indices. Third, other factors that could affect suicide rates, and that might be correlated with the indices examined in this study, were not examined due to the preliminary nature of the current work. Fourth, it is not possible to infer which social and psychological processes were responsible for the observed associations in this study. Fifth, it is possible that other, unknown socioeconomic variables may underlie some of the associations observed in this data set. Sixth, it has been argued that the effect of socioeconomic factors on cross-national variations in suicide rates may be influenced by the prevalence of specific mental disorders, such as depression and alcohol use disorders, which were not evaluated in the current study (Fernquist, 2007). Finally, given the low magnitude of most of the observed associations, the effects of any large-scale suicide prevention programme based solely on social and economic assistance are likely to be modest and to depend on “micro”-level psychological factors.

## Conclusions

Social factors are important determinants of suicide. Despite certain limitations, the results of the current study suggest that sustainable development may play a role in the mitigation of suicide risk, Economic inequality may contribute to variations in suicide risk, particularly in men. Moreover, the strength and direction of the associations between socioeconomic factors and suicide varies across income groups, highlighting the need for an in-depth understanding of each country's social, cultural and economic profile when planning large-scale social interventions aimed at suicide prevention. These results underline the fact that suicide prevention cannot be viewed in purely medical or psychological terms; instead, it must be seen as part of a broader goal of sustainable human development, which involves both environmental sustainability and the equitable distribution of material and financial resources. Based on the associations observed in this study, it can be tentatively suggested that models of human development that balance individual wellbeing with environmental sustainability could reduce the risk of suicide; however, the specific form and content of such models should be adapted to the unique cultural and economic profile of each country.

## Data availability statement

The raw data supporting the conclusions of this article will be made available by the authors, without undue reservation.

## Author contributions

The author confirms being the sole contributor of this work and has approved it for publication.
