# Supplementary material for: The association between nation-level social and economic indices and suicide rates: A pilot study
Source: Front Sociol. 2023 Mar 31;8:1123284. doi: 10.3389/fsoc.2023.1123284 (PMC10102579; doi:10.3389/fsoc.2023.1123284)
Supplement: Supplementary file 1 [file Table_1.pdf]

Supplementary Tables

1. Correlations between national suicide rates and potential confounding factors included in the partial correlation analyses

Var	GDP	Unemp	HDI	Pop	Land	PopDens	Urb	Lat
**Sui (Total)**	-.12 (.099)	-.04 (.589)	-.19 (.012)*	.07 (376)	.22 (.003)**	-.28 (<.001)**	-.09 (.229)	-.01 (.873)
**Sui (Male)**	-.11 (.141)	-.03 (.717)	-.17 (.023)*	.00 (.956)	.17 (.019)*	-.29 (<.001)**	-.14 (.069)	-.02 (.750)
**Sui (Female)**	-.18 (.014)*	-.02 (.769)	-.26 (<.001)**	.13 (.091)	.25 (<.001)**	-.25 (<.001)**	-.14 (.065)	.00 (.993)
**Sui (Male to female ratio)**	.10 (.166)	-.01 (.893)	.14 (.061)	-.16 (.031)*	-.07 (.345)	-.10 (.166)	.04 (.640)	-.04 (.579)

2. Inter-correlations between socioeconomic indices

Var	SWB	HPI	DI	GDI	GII	Gini	LISC
**SWB**	-	.44 (<.001)**	.63 (<.001)**	.50 (<.001)**	-.77 (<.001)**	-.33 (<.001)**	.68 (<.001)**
**HPI**		-	.37 (<.001)**	.21 (.011)*	-.25 (.003)**	-.10 (.254)	.24 (.003)**
**DI**			-	.52 (<.001)**	-.58 (<.001)**	-.17 (.038)*	.50 (<.001)**
**GDI**				-	-.61 (<.001)**	.01 (.904)	.45 (<.001)**
**GII**					-	.50 (<.001)**	-.59 (<.001)**
**Gini**						-	-.30 (<.001)**

3. Correlations between socioeconomic indices and potential confounding factors included in the partial correlation analyses

Var	GDP	Unemp	HDI	Pop	Land	PopDens	Urb	Lat
**SWB**	.84 (<.001)**	-.12 (.130)	.80 (<.001)**	-.12 (.139)	-.13 (.119)	.04 (.673)	.67 (<.001)**	.38 (<.001)**
**HPI**	.22 (.007)**	.06 (.488)	.32 (<.001)**	.08 (.329)	-.05 (.512)	.167 (.042)*	.18 (.030)*	.02 (.796)
**DI**	.65 (<.001)**	.10 (.217)	.62 (<.001)**	-.13 (.092)	-.15 (.058)	.05 (.540)	.37 (<.001)**	.27 (<.001)**
**GDI**	.57 (<.001)**	.00 (.999)	.64 (<.001)**	-.25 (.001)**	-.20 (.009)**	-.01 (.938)	.35 (<.001)**	.21 (.008)**
**GII**	-.87 (<.001)**	.04 (.580)	-.89 (<.001)**	.13 (.099)	.19 (.016)*	-.12 (.142)	-.55 (<.001)**	-.52 (<.001)**
**Gini**	-.32 (<.001)**	.24 (.002)**	-.33 (<.001)**	.02 (.780)	.16 (.045)*	-.23 (.004)**	-.16 (.039)*	-.39 (<.001)**
**LISC**	.62 (<.001)**	-.29 (<.001)**	.56 (<.001)**	-.09 (.280)	-.10 (.208)	.04 (.633)	.32 (<.001)**	.26 (<.001)**

Abbreviations: DI, Democracy Index; GDI, Gender Development Index; GDP, gross domestic product per capita; GII, Gender Inequality Index; Gini, Gini coefficient of income inequality; HDI, Human Development Index; HPI, Happy Planet Index; Land, total land area in square kilometres; Lat, absolute value of the latitude (distance from the equator); LISC, Legatum Index of Social Capital; Pop, total population; PopDens, population density; Sui, suicide rate; SWB, subjective well-being (World Happiness Report score); Unemp, unemployment rate; Urb, percentage of population living in urban areas (urbanization); Var, variable

* significant at $p < .05$; ** significant at $p < .01$

4. Correlations between national suicide rates and socioeconomic indices, sorted by World Bank income groups

a) High-income countries (*n* = 53)

Variable	World Happiness Report score	Happy Planet Index	Democracy Index	Gender Development Index	Gender Inequality Index	Gini coefficient	Legatum Index of Social Capital
**Suicide rate (total)**	.15 (.315)	-.01 (.975)	.44 (.002)**	.07 (.638)	-.40 (.003)**	-.24 (.133)	.14 (.336)
**Suicide rate (male)**	.08 (.600)	.04 (.807)	.48 (.001)**	.13 (.384)	-.36 (.010)*	-.19 (.227)	.06 (.687)
**Suicide rate (female)**	.29 (.045)	.11 (.464)	.56 (<.001)**	.02 (.885)	-.55 (<.001)	-.24 (.129)	.27 (.065)
**Male-to-female suicide ratio**	-.43 (.003)**	-.15 (.324)	-.31 (.035)*	.22 (.129)	.53 (<.001)**	.13 (.430)	-.38 (.007)**

b) High middle-income countries (*n* = 48)

Variable	World Happiness Report score	Happy Planet Index	Democracy Index	Gender Development Index	Gender Inequality Index	Gini coefficient	Legatum Index of Social Capital
**Suicide rate (total)**	-.12 (.474)	-.49 (.002)**	.02 (.905)	.32 (.036)*	-.06(.684)	.23 (.131)	.32 (.041)*
**Suicide rate (male)**	-.12 (.475)	-.46 (.004)**	.05 (.760)	.33 (.026)*	-.05 (.746)	.24 (.129)	.31 (.050)*
**Suicide rate (female)**	-.18 (.289)	-.57 (<.001)**	-.08 (.613)	.23 (.130)	-.12 (.440)	.21 (.187)	.29 (.059)
**Male-to-female suicide ratio**	.07 (.684)	.06 (.724)	.27 (.080)	.29 (.055)	.13 (.416)	.09 (.571)	.07 (.671)

c) Low middle-income countries (*n* = 53)

Variable	World Happiness Report score	Happy Planet Index	Democracy Index	Gender Development Index	Gender Inequality Index	Gini coefficient	Legatum Index of Social Capital

**Suicide rate (total)**	-.39 (.011)*	-.45 (.002)**	.02 (.891)	.13 (.402)	.14 (.346)	.24 (.083)	-.18 (.239)
**Suicide rate (male)**	-.35 (.024)*	-.42 (.005)**	.06 (.705)	.19 (.195)	.14 (.376)	.26 (.068)	-.15 (.314)
**Suicide rate (female)**	-.46 (.003)**	-.46 (.002)**	-.11 (.473)	-.08 (.619)	.15 (.332)	.20 (.153)	-.22 (.151)
**Male-to-female suicide ratio**	.06 (.710)	-.03 (.865)	.26 (.076)	.46 (.001)**	.01 (.928)	.15 (.306)	.06 (.670)

d) Low-income countries (*n* = 28)

**Variable**	**World Happiness Report score**	**Happy Planet Index**	**Democracy Index**	**Gender Development Index**	**Gender Inequality Index**	**Gini coefficient**	**Legatum Index of Social Capital**
**Suicide rate (total)**	.38 (.066)	-.25 (.265)	.08 (.707)	.22 (.296)	.11 (.628)	.48 (.015)*	.41 (.032)*
**Suicide rate (male)**	.35 (.096)	-.16 (.498)	.08 (.689)	.33 (.116)	-.03 (.886)	.57 (.003)**	.39 (.046)*
**Suicide rate (female)**	.39 (.058)	-.41 (.066)	.05 (.802)	-.06 (.794)	.39 (.085)	.16 (.456)	.47 (.013)*
**Male-to-female suicide ratio**	.00 (.987)	.08 (.732)	.05 (.802)	.50 (.013)*	-.47 (.033)*	.62 (<.001)**	-.02 (.931)

***** Significant at $p < .05$; ** Significant at $p < .01$.
