# Supplementary figures and images for: The association between nation-level social and economic indices and suicide rates: A pilot study
Source: Front Sociol. 2023 Mar 31;8:1123284. doi: 10.3389/fsoc.2023.1123284 (PMC10102579; doi:10.3389/fsoc.2023.1123284)

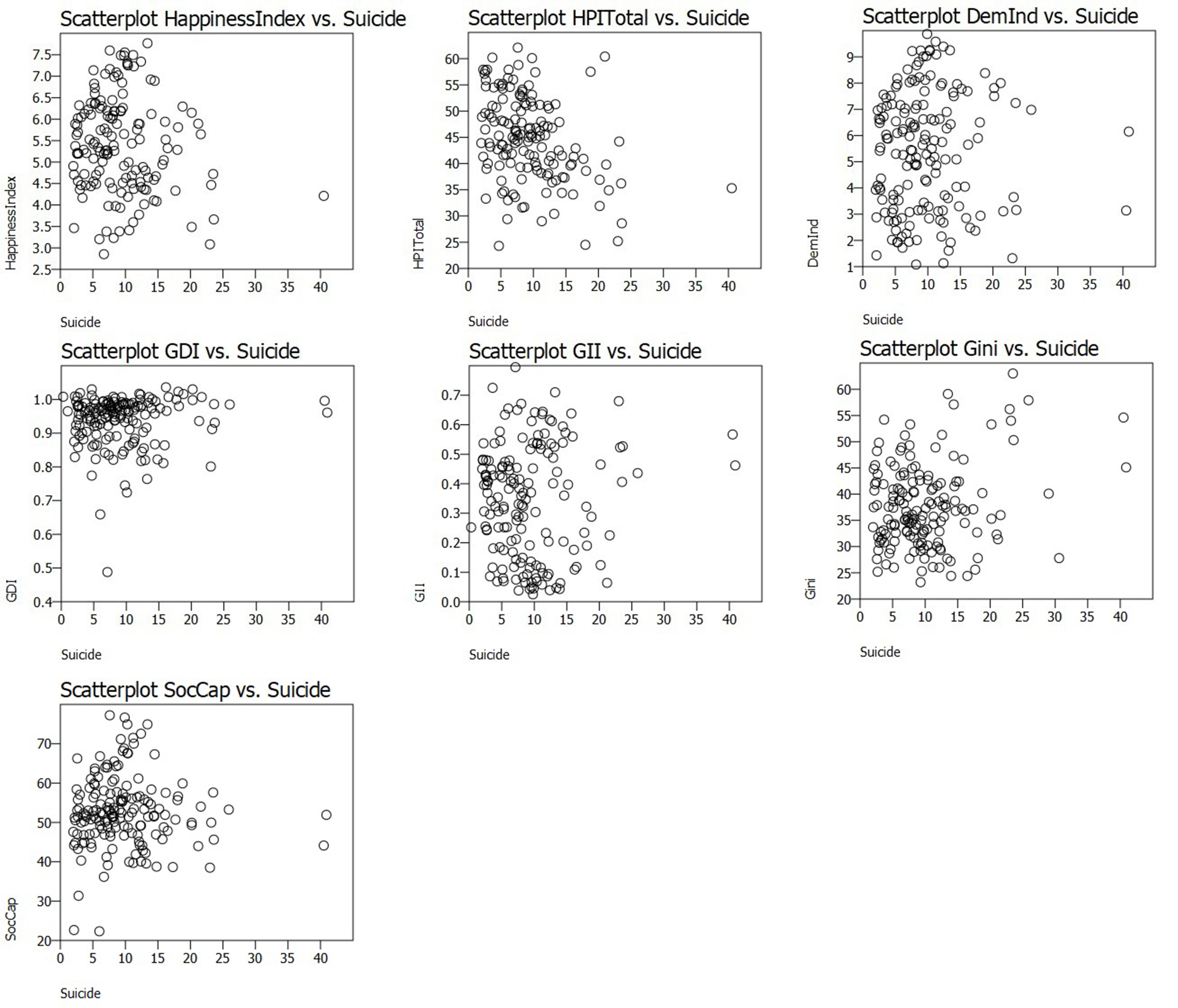

Supplement: Supplementary file 2 [file Image_1.JPEG]
